# Supplementary material for: Water-Soluble Chemical Vapor Detection Enabled by Doctor-Blade-Coated Macroporous Photonic Crystals
Source: Sensors (Basel). 2020 Sep 25;20(19):5503. doi: 10.3390/s20195503 (PMC7582252; doi:10.3390/s20195503)
Supplement: Supplementary file 1 [file sensors-20-05503-s001.pdf]

sensors

MDPI

Article

Water-Soluble Chemical Vapor Detection Enabled by Doctor Blade Coated Macroporous Photonic Crystals

Min-Fang Wu [1], Hui-Ping Tsai [2], Chia-Hua Hsieh [1], Yi-Cheng Lu [1], Liang-Cheng Pan[1] and Hongta Yang [1]*

1 Department of Chemical Engineering, National Chung Hsing University, 145 Xingda Road, Taichung City 40227, Taiwan
2 Department of Civil Engineering, National Chung Hsing University, 145 Xingda Road, Taichung City 40227, Taiwan; huiping.tsai@nchu.edu.tw
* Correspondence:: hyang@dragon.nchu.edu.tw

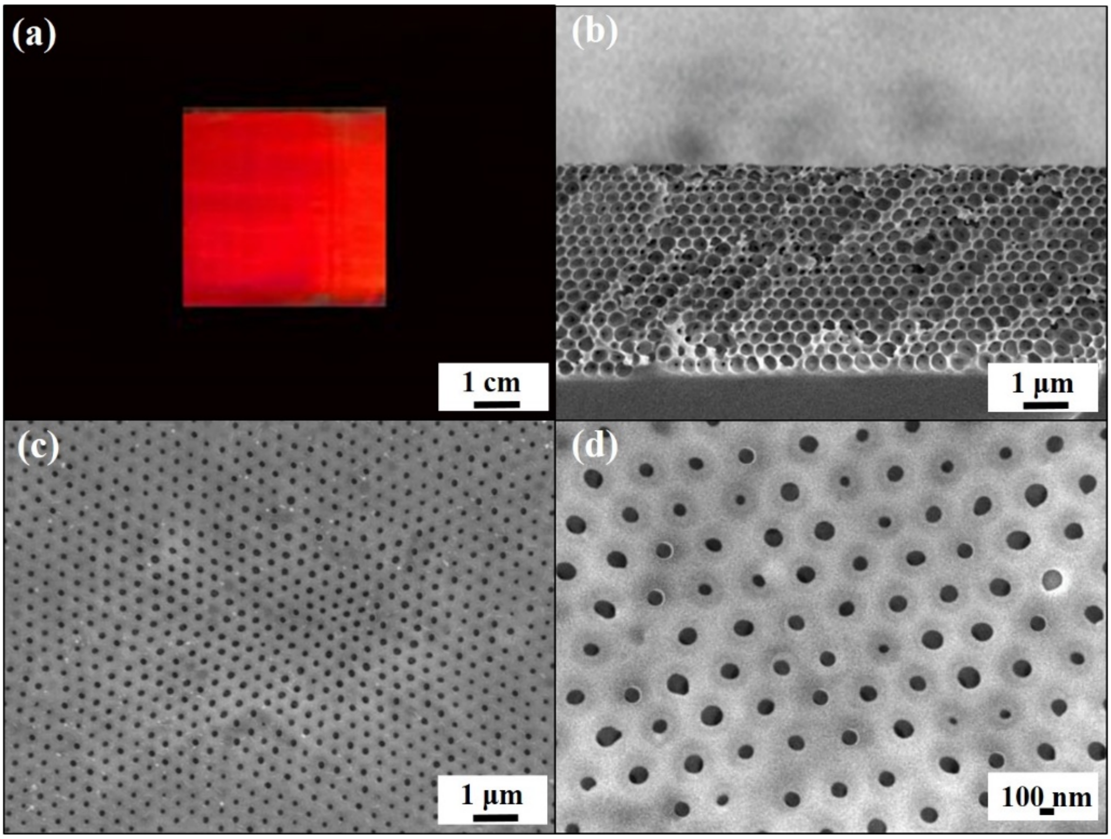

Figure S1. (a) Photographic image of a macroporous poly (2-hydroxyethyl methacrylat)/poly(ethoxylated trimethylolpropane triacrylate) (poly(HEMA)/poly(ETPTA)) film templated from 355 nm silica colloidal crystals. (b) Cross-sectional SEM image and (c) top-view SEM image of the sample in (a). (d) Magnified SEM image of (c).

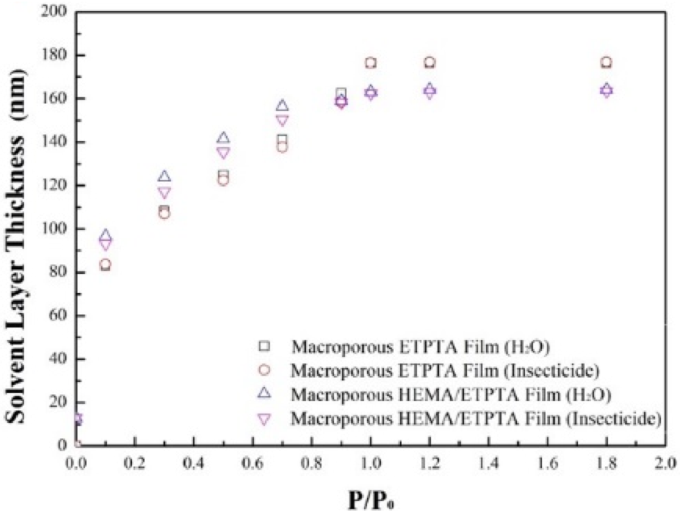

Figure S2. Calculated condensed liquid layer thicknesses of a macroporous poly(ETPTA) film templated from 355 nm silica colloidal crystals and a macroporous poly(HEMA)/poly(ETPTA) film templated from 355 nm silica colloidal crystals under different water vapor pressures and insecticide vapor pressures using a gravimetric analysis.

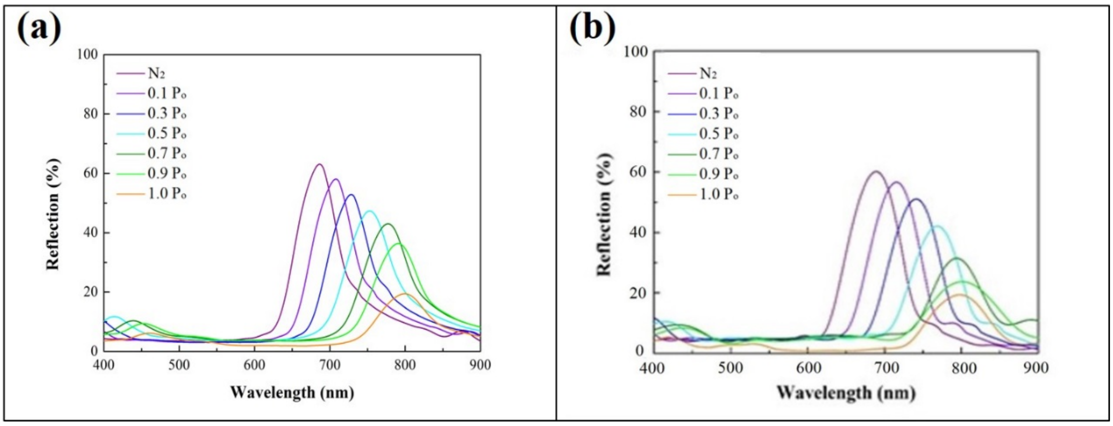

Figure S3. Normal-incidence specular reflection spectra obtained from a macroporous poly(HEMA)/poly(ETPTA) film templated from 355 nm silica colloidal crystals under different insecticide vapor pressures using (a) diluted commercial insecticide containing 0.01 vol. % chemicals and (b) diluted commercial insecticide containing 0.001 vol. % chemicals. P_0 represents the saturation insecticide vapor pressure at 28 °C.

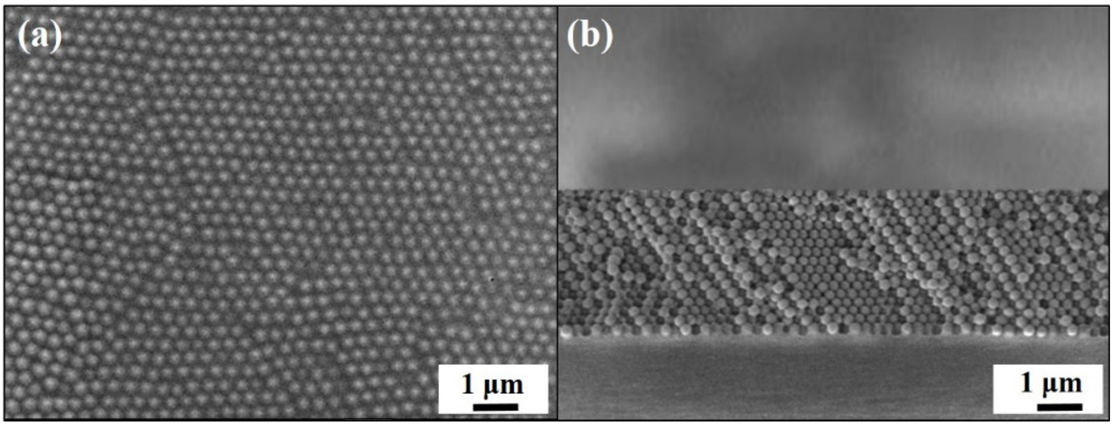

Figure S4. (a) Top-view SEM image of a doctor blade coated 250 nm silica colloidal crystal/poly(ETPTA) composite. (b) Cross-sectional SEM image of the sample in (a).

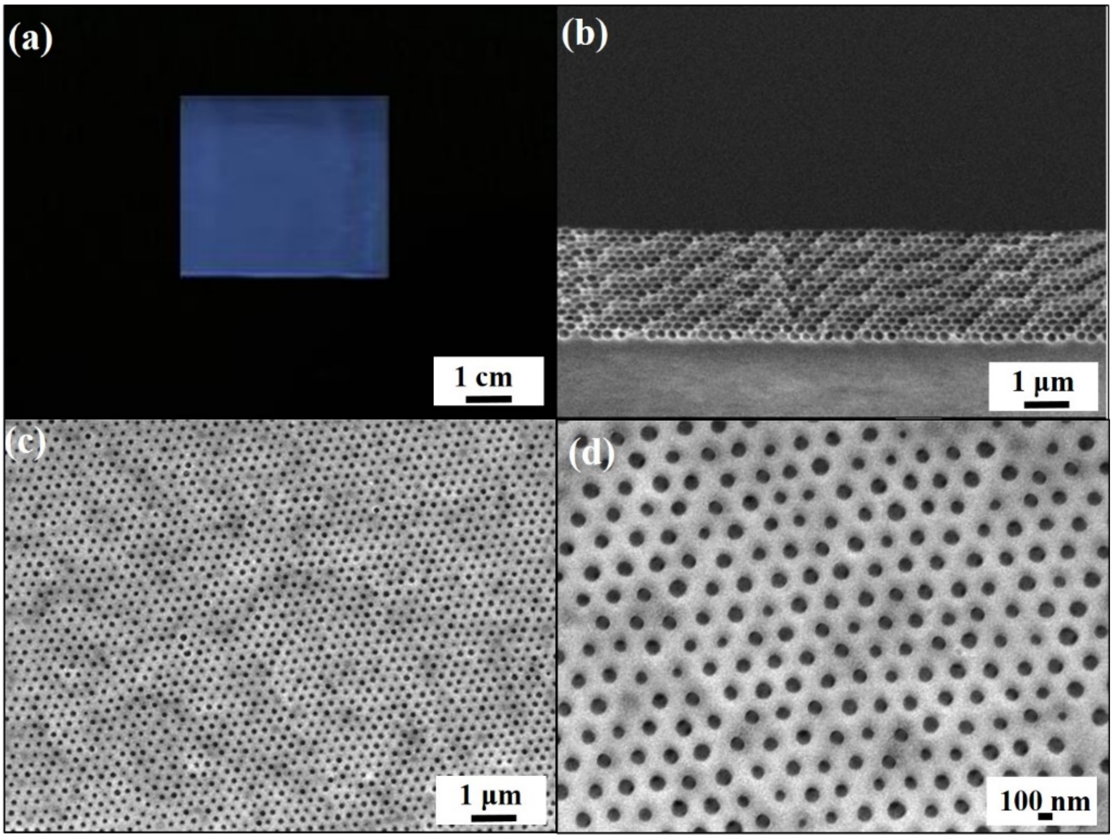

Figure S5. (a) Photographic image of a macroporous poly(ETPTA) film templated from 250 nm silica colloidal crystals. (b) Cross-sectional SEM image and (c) top-view SEM image of the sample in (a). (d) Magnified SEM image of (c).

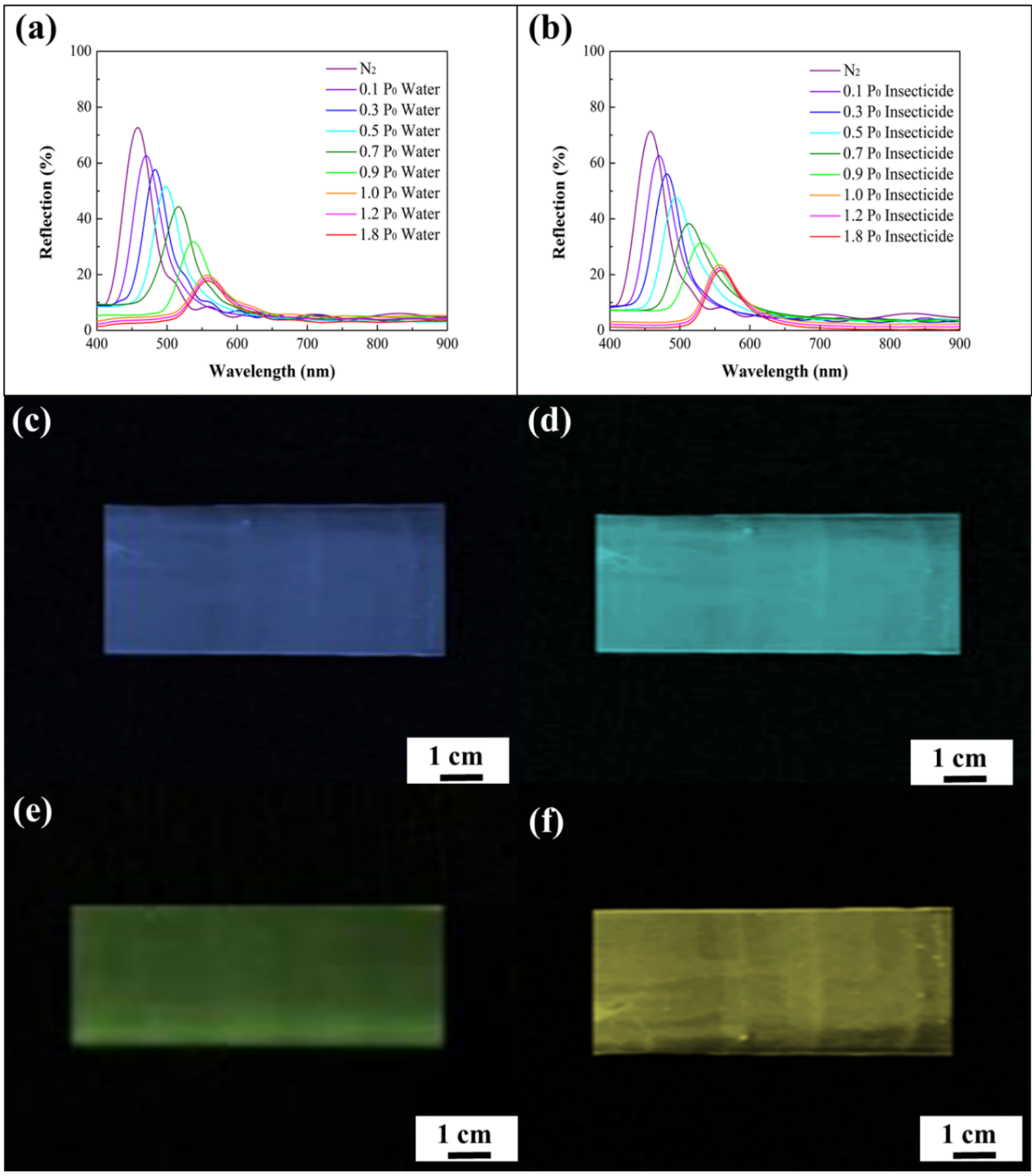

Figure S6. Normal-incidence specular reflection spectra obtained from a macroporous poly(ETPTA) film templated from 250 nm silica colloidal crystals under (a) different water vapor pressures and (b) different insecticide vapor pressures. Photographic images obtained from the macroporous film under different insecticide vapor pressures. (c) 0 P_0; (d) 0.3 P_0; (e) 0.7 P_0; (f) 1.0 P_0. P_0 represents the saturation insecticide vapor pressure at 28 °C.

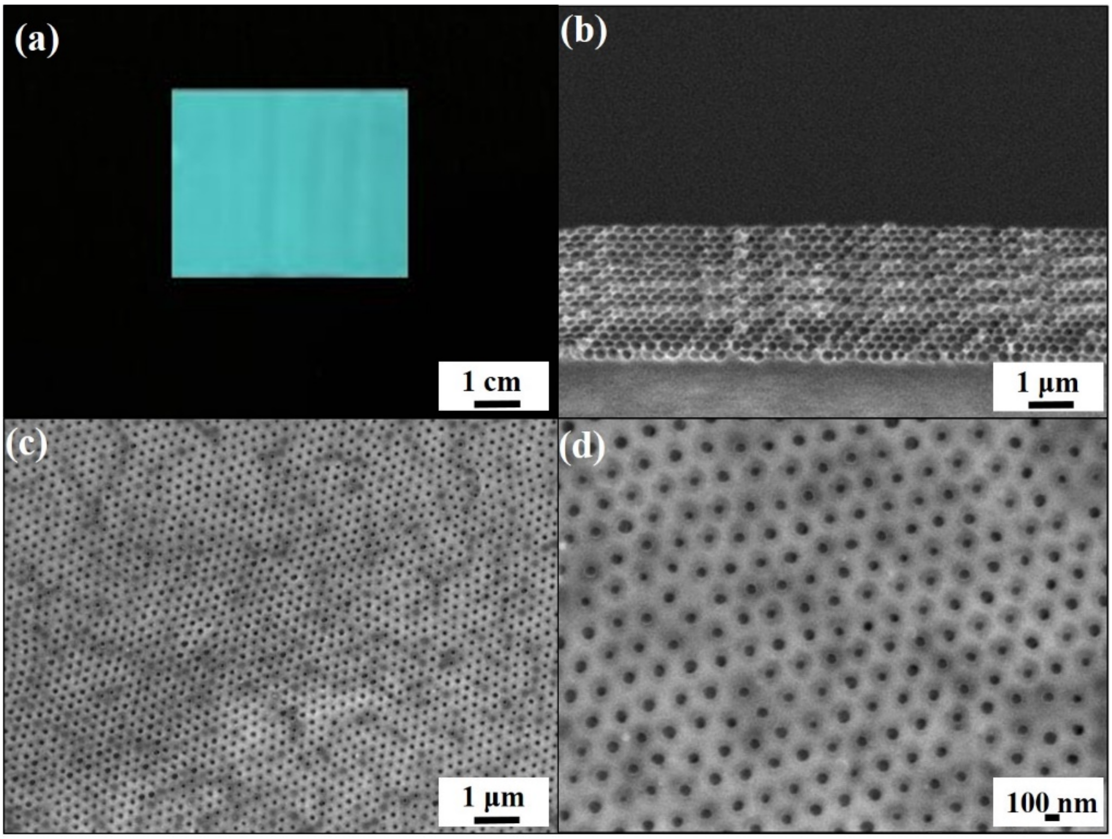

Figure S7. (a) Photographic image of a macroporous poly(HEMA)/poly(ETPTA) film templated from 250 nm silica colloidal crystals. (b) Cross-sectional SEM image and (c) top-view SEM image of the sample in (a). (d) Magnified SEM image of (c).

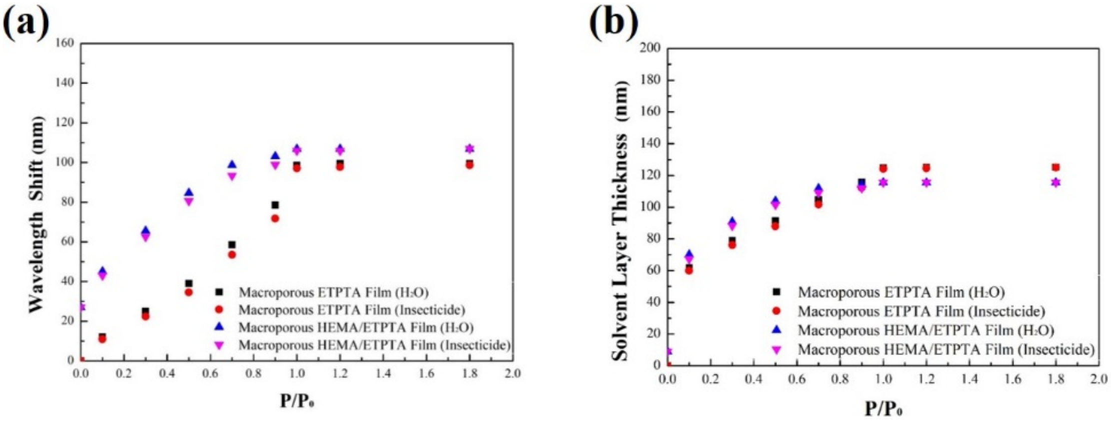

Figure S8. (a) The reflection peak position shifts of a macroporous poly(ETPTA) film templated from 250 nm silica colloidal crystals and a macroporous poly(HEMA)/poly(ETPTA) film templated from 250 nm silica colloidal crystals under different vapor pressures. (b) Calculated condensed liquid layer thicknesses of the macroporous films under different vapor pressures.

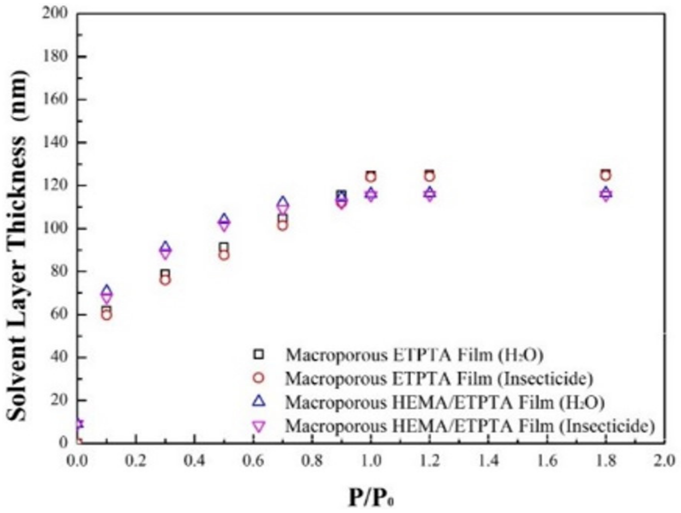

Figure S9. Calculated condensed liquid layer thicknesses of a macroporous poly(ETPTA) film templated from 250 nm silica colloidal crystals and a macroporous poly(HEMA)/poly(ETPTA) film templated from 250 nm silica colloidal crystals under different water vapor pressures and insecticide vapor pressures using a gravimetric analysis.

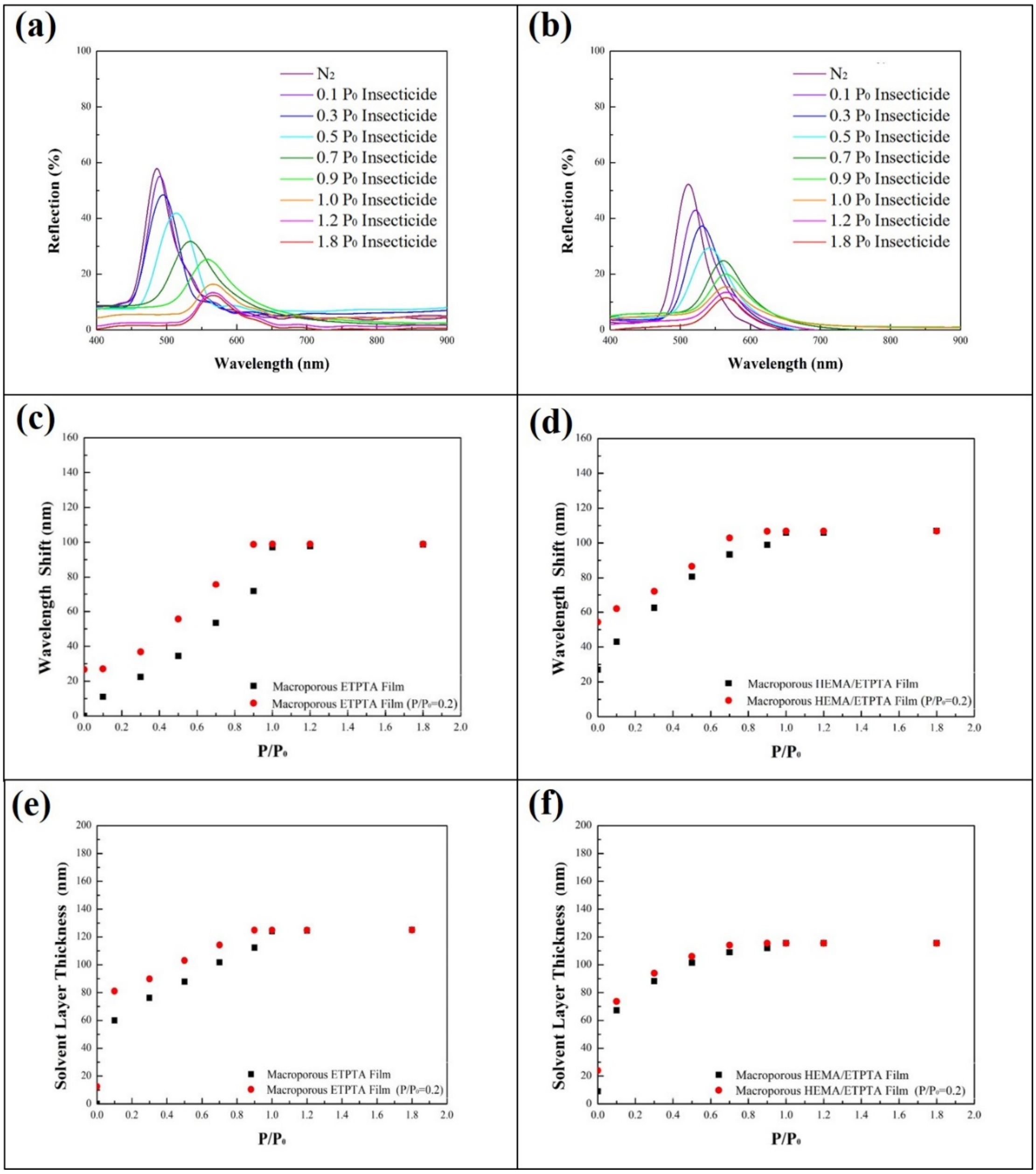

Figure S10. Normal-incidence specular reflection spectra obtained from (a) a macroporous poly(ETPTA) film templated from 250 nm silica colloidal crystals and (b) a macroporous poly(HEMA)/poly(ETPTA) film templated from 250 nm silica colloidal crystals under a fixed water vapor pressure (0.2 P_0) and different insecticide vapor pressures. P_0 represents the saturation water vapor pressure at 28 °C. The corresponding reflection peak position shifts of (c) the macroporous poly(ETPTA) film and (d) the macroporous poly(HEMA)/poly(ETPTA) film under a fixed water vapor pressure (0.2 P_0) and different insecticide vapor pressures. The corresponding calculated condensed liquid layer thicknesses of (e) the macroporous poly(ETPTA) film and (f) the macroporous poly(HEMA)/poly(ETPTA) film under a fixed water vapor pressure (0.2 P_0) and different insecticide vapor pressures.
